# Comprehensive analyses of the citrus WRKY gene family involved in the metabolism of fruit sugars and organic acids

**DOI:** 10.3389/fpls.2023.1264283

**Published:** 2023-09-15

**Authors:** Mingfei Zhang, Wen Lu, Xinxia Yang, Qin Li, Xingyu Lin, Kexin Liu, Chunmei Yin, Bo Xiong, Ling Liao, Guochao Sun, Siya He, Jiaxian He, Xun Wang, Zhihui Wang

**Affiliations:** College of Horticulture, Sichuan Agricultural University, Chengdu, Sichuan, China

**Keywords:** WRKY gene family, citrus, fruit quality, sugar, organic acid, expression network

## Abstract

Sugars and organic acids are the main factors determining the flavor of citrus fruit. The WRKY transcription factor family plays a vital role in plant growth and development. However, there are still few studies about the regulation of citrus WRKY transcription factors (CsWRKYs) on sugars and organic acids in citrus fruit. In this work, a genome-wide analysis of *CsWRKY*s was carried out in the citrus genome, and a total of 81 *CsWRKY*s were identified, which contained conserved WRKY motifs. *Cis*-regulatory element analysis revealed that most of the *CsWRKY* promoters contained several kinds of hormone-responsive and abiotic-responsive *cis*-elements. Furthermore, gene expression analysis and fruit quality determination showed that multiple *CsWRKY*s were closely linked to fruit sugars and organic acids with the development of citrus fruit. Notably, transcriptome co-expression network analysis further indicated that three *CsWRKY*s, namely, *CsWRKY3*, *CsWRKY47*, and *CsWRKY46*, co-expressed with multiple genes involved in various pathways, such as Pyruvate metabolism and Citrate cycle. These CsWRKYs may participate in the metabolism of fruit sugars and organic acids by regulating carbohydrate metabolism genes in citrus fruit. These findings provide comprehensive knowledge of the *CsWRKY* family on the regulation of fruit quality.

## Introduction

Citrus fruit is one of the largest fruit crops in the world and is rich in various nutrients, such as sugars, organic acids, amino acids, carotenoids, and flavonoids (Guo et al., 2016; Sheng et al., 2017). Citrus fruit flavor is mainly determined by the content of sugars and organic acids and is one of the important factors affecting consumer purchases (Lin et al., 2015). The main components of soluble sugars in citrus fruit are sucrose, fructose, and glucose (Wu et al., 2021), and citric acid is the decisive organic acid, which accounts for 70% to 90% of organic acids in citrus fruit (Sheng et al., 2017). Studies on fruit quality reveal that the metabolism of sugars and organic acids is regulated by different genes, such as sucrose phosphate synthase (SPS), citrate synthetase (CS), and isocitrate dehydrogenase (IDH) (Etienne et al., 2013; Ruan, 2014; Guo et al., 2016; Hussain et al., 2017; Wan et al., 2018; Zhang et al., 2021). Notably, the metabolism of sugars and organic acids is reported to be regulated by multiple transcription factors (TFs), such as WRKYs, MYBs, and ERFs. Previously, CitERF16 was found to promote sucrose accumulation by activating the expression of *CitSWEET11d*, and CitZAT5 regulated the accumulation of hexose by mediating the expression of *CitSUS5* and *CitSWEET6* (Hu et al., 2021; Fang et al., 2023). Moreover, R3-MYB transcription factor TRIPTYCHON-LIKE acted as a repressor of the accumulation of citric acid and anthocyanin, and CitWRKY1 and CitERF6 participated in citric acid degradation of citrus fruit (Li et al., 2017; Li et al., 2020; He et al., 2022).

WRKY TFs are known as one of the largest families of transcriptional regulators in plants and are identified to bind to W-box [(C/T)TGAC(C/T)] *cis*-elements in the promoter of their target genes (Ciolkowski et al., 2008; Rushton et al., 2010). So far, a large number of WRKY genes have been identified in plants, and genome-wide analyses of WRKYs are also conducted in multiple species. The WRKY proteins always contain one or two conserved WRKYGQK motifs at the N-terminus and a typical zinc-finger motif (C2H2 and C2HC) at the C-terminus (Rushton et al., 1995; Eulgem et al., 2000). Meanwhile, WRKYs are further divided into three groups (I, II, and III) based on their WRKY domains and zinc-finger motifs (Mangelsen et al., 2008). Plant WRKY proteins are well-known to be involved in various stress responses and plant metabolite biosynthesis (Rushton et al., 2010; Han et al., 2021b; Wang et al., 2023b).

Recently, multiple studies have revealed that members of WRKY TFs are widely involved in different processes throughout the growth and development of plants. For example, *Arabidopsis* WRKYs (such as *AtWRKY18* and *AtWRKY63*) were also found to regulate seed germination and root growth involved in hormone signaling (Chen et al., 2010; Ren et al., 2010). The mutations of *AtWRKY12*, *OsWRKY36*, and *OsWRKY102* led to the accumulation of secondary cell wall and stem biomass, thereby changing the stem morphology (Yu et al., 2013; Miyamoto et al., 2020). Meanwhile, heterologous overexpression of *CpWRKY71*, *GmWRKY58*, and *GmWRKY76* induced the expression of flowering-promoting genes (including *FT*, *LFY*, and *FUL*) and promoted flowering in *Arabidopsis* (Yang et al., 2016; Huang et al., 2019). Notably, several WRKY members were determined to participate in the formation of fruit quality. MaWRKY49 positively regulated pectate lyase genes to modulate fruit ripening (Liu et al., 2023). VvWRKY22 and CdWRKY2 directly regulated the expression of sugar-related genes to mediate sugar biosynthesis (Huang et al., 2021; Huang et al., 2022). Meanwhile, AtWRKY46 and SlWRKY42 were proven to bind the upstream W-box of *ALMT9* and alter malate accumulation (Ding et al., 2013; Ye et al., 2017).

In citrus, several WRKY TFs were reported to regulate the biotic and abiotic stresses, but only a few members were found to take part in the metabolism of fruit quality. Hence, additional studies are required to identify more WRKY TFs linked to fruit quality building. In this study, a comprehensive analysis was performed to identify and characterize WRKY TFs in citrus genomes. Additionally, RNA-Seq data and gene expression were further analyzed to study the potential function of WRKY TFs in the metabolism of sugars and organic acids during citrus fruit development. Our results will provide new insights into the regulation of citrus sugar and organic acid metabolism pathways.

## Materials and methods

### Identification and characteristic analysis of the WRKY gene family

Identification of citrus WRKY genes was performed according to previous studies (Hu et al., 2015; He et al., 2016). The genomic sequences of different citrus species (*Citrus clementina* v1.0, *Citrus grandis* ‘Wanbaiyou’ v1.0, *Poncirus trifoliata* v1.0, and *Citrus sinensis* v2.0) were obtained from the Citrus Pan-genome to Breeding Database (CPBD: http://citrus.hzau.edu.cn/index.php). The *Arabidopsis* WRKY gene members and their protein sequences were obtained from the *Arabidopsis* Information Resource (TAIR: https://www.arabidopsis.org/browse/genefamily/index.jsp). *C. sinensis* WRKY (CsWRKY) transcription factors were identified using HMMER software version 3.0 and the PFAM protein family database using the WRKY domain (PF03106) as a query (Eddy, 2011; Mistry et al., 2021). Further, BLASTP was constructed to validate all non-overlapping WRKY genes.

The chromosome location image of *CsWRKY*s was generated using MapGene2Chrom software (MG2C_v2.1). The exon/intron structure of all *CsWRKY*s was displayed using the TBtools software. The relative molecular weight (RMW) and isoelectric point (pI) of CsWRKY proteins were analyzed using ProtParam (http://web.expasy.org/protparam/).

### Analysis of gene structure and conserved motifs of CsWRKYs

The conserved motifs of WRKY proteins were detected with the multiple EM for motif elicitation (MEME software) (Brown et al., 2013). The optimum width of each motif ranged from 6 to 20, the maximum number of motifs to search was 8, and other parameter settings were default values (Bailey et al., 2006). All candidate WRKYs were further validated by using the National Center for Biotechnology Information (NCBI) Conserved Domain Database (CCD) to ensure that they contained the WRKY domains. The WRKY domains and zinc-finger motifs were predicted based on multiple sequence alignment with BioEdit.

### Phylogenetic relationship analysis of CsWRKY gene family

Multiple sequence alignments of *C. sinensis* and *Arabidopsis thaliana* WRKY protein sequences were performed using molecular evolutionary genetics analysis (MEGA) version 6.0 with 1,000 bootstrap replications, pairwise deletion, and Poisson model. Subsequently, neighbor-joining phylogenetic trees were constructed.

### RNA-Seq data and qRT-PCR analysis

RNA-Seq data were obtained from published data (Wang et al., 2017; Feng et al., 2021). The data that support the findings of this study have been deposited in the NCBI BioProject database under accession numbers PRJNA517400 and PRJNA387319. The method used for RNA-Seq data analysis was performed as described in a previous study (Pertea et al., 2016). In brief, raw reads of RNA-Seq were preprocessed for quality using the FastQC software, and low-quality reads (q < 20) and adapters were trimmed using Trimmomatic. Next, the obtained reads were aligned to the *C. sinensis* genome using HISAT2 with default parameters, and the mapped reads were further assembled by StringTie. Fragments per kilobase per million mapped fragments (FPKM) was used to represent the gene expression levels. The expression profiles of *CsWRKY*s were extracted from the analyzed RNA-Seq data for further study.

Isolation of total RNA from different tissues was carried out according to the procedure reported by Liu et al. (Liu et al., 2007). Specific primer pairs for amplification of *CsWRKY*s were designed by using the Primer Express software (Applied Biosystems, Foster City, CA, USA). The specificity and amplification efficiency of the primers were validated by BLASTN in the sweet orange genome. With the citrus β-actin gene as the internal reference gene, relative gene expression values were calculated using the 2^−ΔΔCt^ method (Livak and Schmittgen, 2001). The sequences of RT-PCR primers are displayed in **Table S7**. All expression data were processed by the Z-score standardization method.

### Co-expression network analysis

Weighted gene co-expression network analysis (WGCNA) (v1.71) package in R was used to construct the co-expression networks (Langfelder and Horvath, 2008). From the total of 29,138 genes, 16,961 genes with a sum FPKM of all samples lower than 1 were removed, and the reserved genes were used for the WGCNA co-expression network analysis. The one-step network construction and module detection function were conducted using an unsigned type of topological overlap matrix (TOM) with a soft-thresholding power β of 14 (R^2^ > 0.9), a minimal module size of 30, and a branch merge cut height of 0.25. The co-expression network of candidate CsWRKYs was visualized by Cytoscape (version 3.6.1) (Shannon et al., 2003).

### Plant materials

‘Newhall’ navel orange (*C. sinensis* Osbeck) fruits were harvested from a commercial orchard in Liangshan Yi autonomous prefecture, Sichuan province, China. Fruits with uniform size and free of visible injury were harvested in August (Aug), October (Oct), November (Nov), and December (Dec). After the harvest, the fruits without obvious damage were transported to the laboratory for further experiments. The pulp was sampled, frozen, homogenized in liquid nitrogen, and kept at −80°C for later analyses. Three replications each containing six fruits were analyzed, and measurements were performed.

### Fruit quality determination

‘Newhall’ navel orange fruits at each stage were chosen for determination of the total soluble solid (TSS) and titratable acid (TA). At each development stage, more than 18 fruits were used for quality determination with three replications. TSS (%) and TA (%) were measured by a digital acidity meter (Pocket PAL-BXIACID1, ATAGO, Tokyo, Japan) following the manufacturer’s instructions.

### Statistical analysis

All data are shown as means ( ± SD) of one representative experiment. Significant differences between different samples were determined using ANOVA followed by Tukey’s test. The heatmaps were performed using R studio software by the pheatmap package. The correlation analysis was performed using R studio software. Figures were drawn using GraphPad Prism (GraphPad Software, CA, USA).

## Results

### Identification and phylogenetic analysis of CsWRKYs

**Figure 1:**
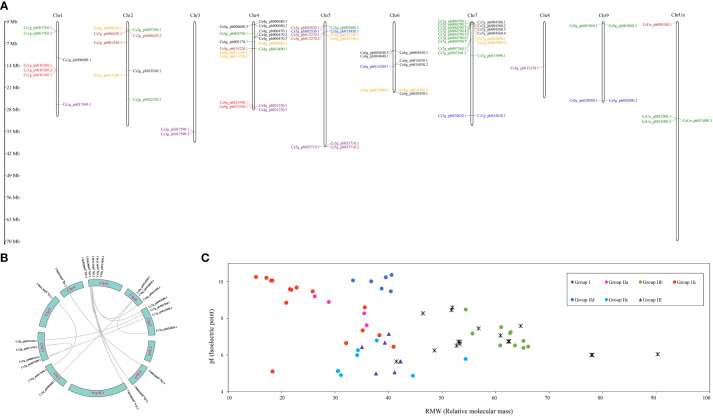
Characterizations of WRKY gene family in *Citrus sinensis*. **(A)** The chromosome distribution of WRKY gene family. **(B)** Repeated events of *CsWRKY* in *C. sinensis* genome. **(C)** Protein properties of CsWRKYs.

**Figure 2:**
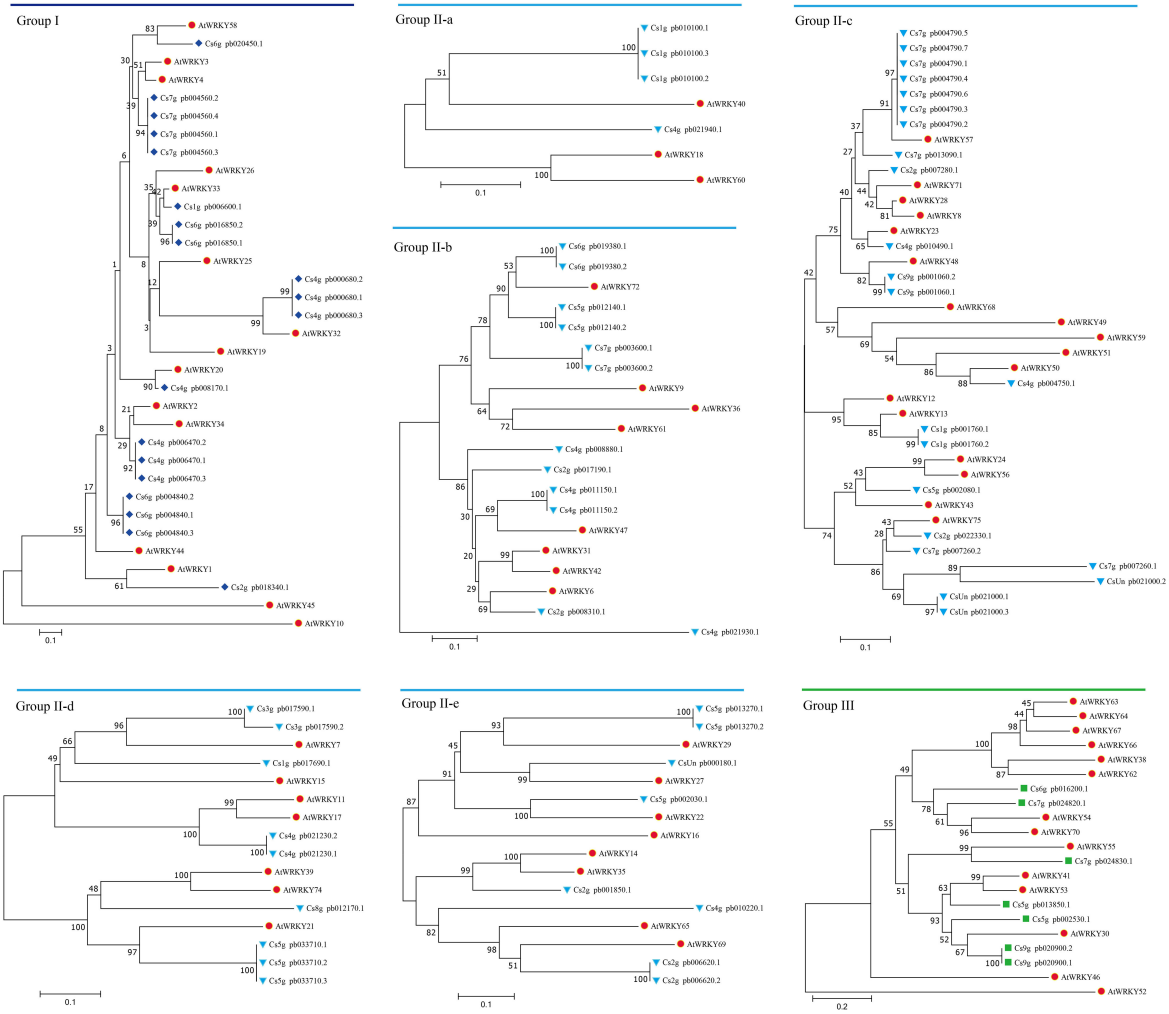
Phylogenetic relationship of WRKY proteins among *Citrus sinensis* and *Arabidopsis thaliana*. Phylogenetic analysis was performed using the neighbor-joining method in MEGA v5.1, and phylogenetic trees were constructed from the conserved WRKY domains of citrus and *Arabidopsis* WRKY proteins. WRKY proteins in *A. thaliana* were represented by red circles. Citrus WRKY proteins were divided into three groups (I, II, and III, which were represented by blue diamonds, blue triangles, and green squares, respectively). Group II included five subgroups (IIa, IIb, IIc, IId, and IIe).

Genome-wide identification of WRKYs was performed in the *C. sinensis* genome. After the removal of redundant sequences and those sequences lacking WRKY motifs, 81 unique CsWRKY transcripts belonging to 47 genes were finally obtained. Consequently, phylogenetic analysis with protein sequences was constructed together with the orthologue WRKYs from the genomes of *A. thaliana* (**Figure 1**). All CsWRKYs were clustered into three distinct groups (group I, group II, and group III), and members of group II were further clustered into five subgroups (group IIa, group IIb, group IIc, group IId, and group IIe). Among these WRKYs, 19 proteins were grouped into group I, 55 proteins were grouped into group II, and seven proteins were grouped into group III (**Figure 2** and **Table S1**). Furthermore, multiple sequence alignments indicated that all CsWRKYs contained the conserved WRKYGQK domains and the C2H2 or C2HC zinc finger-like motifs (**Figure S1**). Most of group I WRKYs were found to have two WRKYGQK domains, and only group III had C2HC zinc finger-like motifs (**Figures S1A, G**). Meanwhile, the corresponding WRKYs were also identified in *C. clementina* (47), *C. grandis* (53), *P. trifoliata* (50), *Atalantia buxifolia* (52), *Fortunella hindsii* (53), and *Citrus ichangensis* (50) (**Table S1**).

### Chromosomal location, putative pI, and RMW of CsWRKYs

To determine *CsWRKY*s’ physical locations, all 81 *CsWRKY*s were mapped onto the chromosomes in the *C. sinensis* genome sequence (**Figure 1A**). Their distribution on chromosomes greatly varied from one in Chr8 to 18 in Chr7. All chromosomes showed the presence of at least one group of *CsWRKY*s, and only group IId WRKYs were localized in Chr3 and Chr8. Meanwhile, four chromosomes (Chr1, Chr2, Chr4, Chr5, and Chr7) were found to contain not less than four WRKY groups. Fourteen pairs of repetitive fragments in the *CsWRKY*s were identified, and three of them had two repeats (**Figure 1B**).

The RMW of the predicted 81 CsWRKY proteins ranged from 15.22 to 90.29 kDa, and the pI ranged from 4.87 to 10.37 (**Figure 1C**; **Table S1**). Notably, the RMW of CsWRKY proteins in groups I (41.25 to 90.29 kDa) and IIb (54.42 to 66.08 kDa) was much bigger than that of other members, while most of CsWRKY proteins (10/18) in group IIc showed smaller RMW, which was less than 26 kDa. The pI of all CsWRKY proteins in group IId ranged from 9.47 to 10.37, while the pI of CsWRKY proteins in groups IIe and III was less than 8.

### Protein motif, gene sequence, and promoter analysis of CsWRKYs

**Figure 3:**
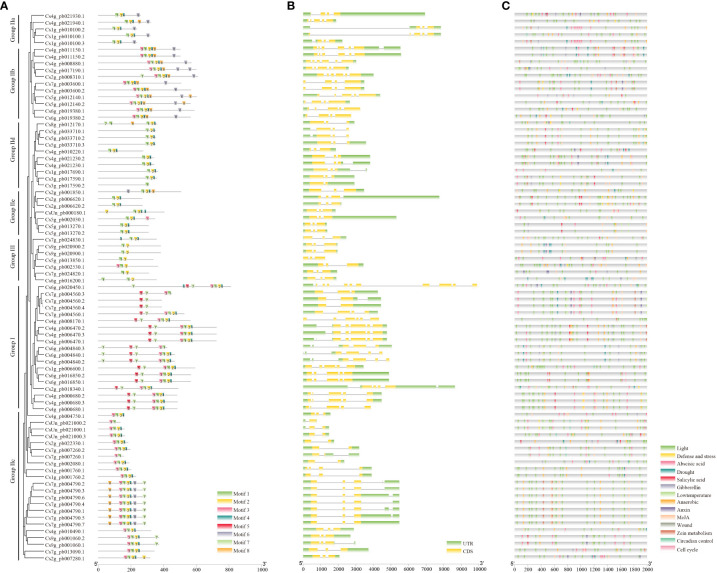
The conserved motifs, gene structure, and predicted *cis*-elements of *CsWRKY*s. **(A)** The conserved motifs, numbers 1–8, are shown in different colored boxes. The details of each motif are provided in **Table S3**. **(B)** The exon–intron structure of *CsWRKY*s. Green boxes represent 5′ UTR and 3′ UTR, yellow boxes represent exons, and gray lines present introns. **(C)** Predicted *cis*-elements in the *CsWRKY* prompters. Different *cis*-elements are represented by different colors.

Furthermore, motif analysis was carried out with the MEME web server, resulting in the discovery of a total of eight conserved motifs in CsWRKY proteins (**Figure 3A**; **Table S2**). The results indicated that all CsWRKY proteins shared similar motifs, especially motifs 1 and 2, which contained the WRKYGQK domain and part of C2H2 or C2HC. Motif 5 was a unique motif of group I proteins, and motifs 6 and 8 were mainly distributed in group IIb and group IIc proteins. Notably, motif 4 was missing in group III proteins. The distribution of conserved motifs is consistent with the phylogenetic tree result.

The exon–intron structures of *CsWRKY*s were further analyzed. Generally, most of *CsWRKY*s (78/81) contained two to five exons (**Figure 3B**). Most of the *CsWRKY*s in the same group have similar intron–exon structures. For example, *CsWRKY*s (17/19) in group I contained four to five exons, and only two genes had 10 or six exons. Moreover, seven *CsWRKY*s in group III showed three exons and two introns. However, one member of the group I *CsWRKY*s had 11 exons, while group IIc *CsWRKY*s had only one exon.

To analyze the potential *cis*-elements, the 2-kb sequences upstream of the translation start sites in each *CsWRKY* were submitted to the online tool PlantCARE. A total of 14 types of *cis*-elements associated with responses to different hormones, external environmental signals, cell cycle (MSA-like), circadian control, and zein metabolism were finally identified (**Figure 3C**; **Table S3**). Our results indicated that the light-responsive element existed in the promoters of all *CsWRKY*s. Meanwhile, the promoters of most *CsWRKY*s were found to have the abscisic acid-responsive element (64/81), anaerobic regulatory elements (70/81), and MeJA-responsive element (70/81). The drought-responsive elements, gibberellin-responsive elements, low-temperature-responsive elements, and zein metabolism regulatory elements exist in nearly one-third of *CsWRKY*s.

### Expression profiles of *CsWRKY*s in the pulp of citrus fruit

**Figure 4:**
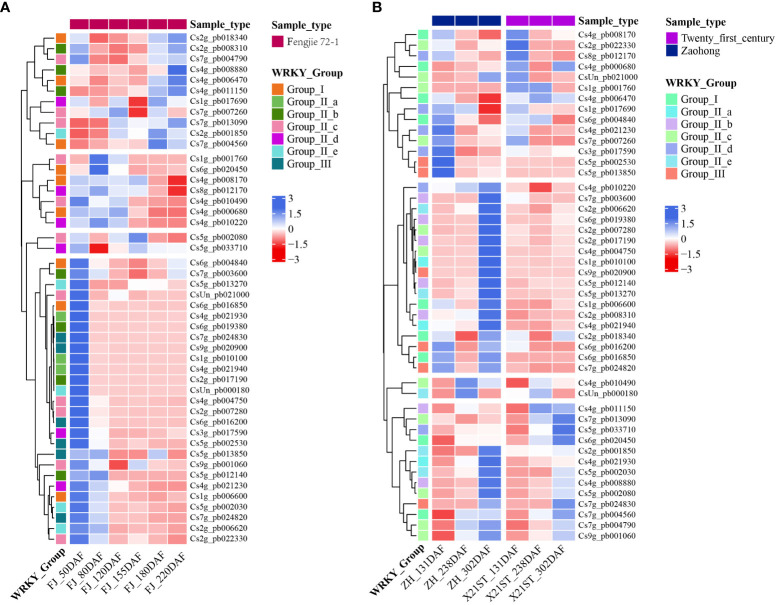
Expression of *CsWRKY*s in different development stages of citrus fruit. The transcription levels of the *CsWRKY*s in the pulp of ‘Fengjie 72-1’ **(A)**, ‘Zaohong’, and ‘Twenty-first century’ **(B)** orange fruits at different development stages. Different *CsWRKY* groups are represented by different colors. The color scale shows increasing expression levels from red to blue.

To further explore the expression pattern of *CsWRKY* genes in citrus fruit, the expression of 47 *CsWRKY* genes at different fruit development stages based on publicly available transcriptomic data was analyzed. The results showed that all *CsWRKY*s had different expression patterns among different varieties (**Figure 4**). In the pulp of ‘Fengjie 72-1’ fruit (**Figure 4A**), most *CsWRKY*s (27/47) were highly expressed at 50 days after flowering (DAF) and showed an overall downregulated expression pattern with fruit ripening. Several genes showed high transcription levels in young fruit (50–120 DAF). Moreover, the transcript level of some *CsWRKY*s (11/47) was generally upregulated with the development of fruit, but two of them showed the lowest transcript level during the expansion period (155 DAF). In the pulp of ‘Zaohong’ and ‘Twenty-first century’ orange fruits, the expression patterns of all *CsWRKY*s are divided into two groups. As shown in **Figure 4B**, the transcript abundance of most *CsWRKY*s (29/47) was much higher at 302 DAF in both ‘Zaohong’ and ‘Twenty-first century’ orange fruits and dramatically decreased with fruit development. Notably, many of these genes (25/29) had higher expression levels in the pulp of ‘Zaohong’ orange fruit. Meanwhile, some *CsWRKY*s showed high expression at 131 DAF, while a significant reduction was observed with fruit development.

### Correlation analysis between *CsWRKY*s and sugars and organic acids in citrus fruit

**Figure 5:**
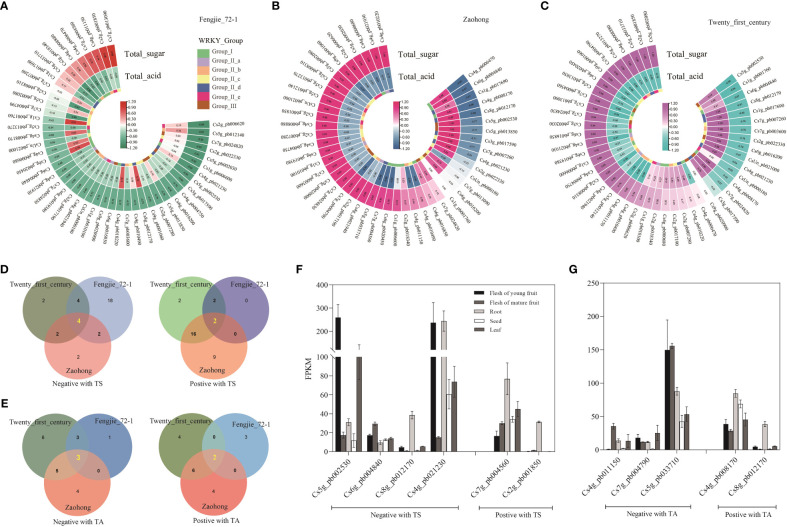
Correlation analysis of *CsWRKY*s with sugars and organic acids in citrus fruit. Heatmap of Pearson’s correlation coefficients between *CsWRKY*s and sugar and acid contents in the pulp of ‘Fengjie 72-1’ **(A)**, ‘Zaohong’ **(B)**, and ‘Twenty-first century’ **(C)** orange fruits. Venn diagrams of *CsWRKY*s co-expressing with the content of total sugar **(D)** and total organic acid **(E)** (correlation coefficient >0.6 or <−0.6). The expression profile of co-expressing *CsWRKY*s in different tissues of orange fruit **(F, G)**.

To further identify the potential functional *CsWRKY*s involved in the formation of fruit quality, Pearson’s correlation coefficients between *CsWRKY* expression and sugar and organic acid contents were analyzed. In different citrus varieties, the number of *CsWRKY*s related to sugars and organic acids was different (**Figures 5A–C**). Many *CsWRKY*s were negatively correlated with sugar content in ‘Fengjie 72-1’ fruit, but a number of them showed a positive relationship with sugars in ‘Zaohong’ and ‘Twenty-first century’ fruits. Meanwhile, few genes had a relatively strong relationship (correlation coefficient >0.5 or <−0.5) with the content of organic acids in ‘Fengjie 72-1’ fruit, but more genes were strongly correlated in ‘Zaohong’ and ‘Twenty-first century’ fruits. *CsWRKY*s with a strong relationship (correlation coefficient >0.6 or <−0.6) with sugars and organic acids were selected for Venn diagram analysis. Four *CsWRKY*s (*Cs5g_pb002530*, *Cs6g_pb004840*, *Cs8g_pb012170*, and *Cs4g_pb021230*) were negatively correlated with sugars, while two (*Cs7g_pb004560* and *Cs2g_pb001850*) were positively correlated (**Figure 5D**). Moreover, three *CsWRKY*s (*Cs4g_pb011150*, *Cs7g_pb004790*, and *Cs5g_pb033710*) were found to be negatively correlated with organic acids, while two (*Cs4g_pb008170* and *Cs8g_pb012170*) were positively correlated (**Figure 5E**). *Cs8g_pb012170* was found to be positively correlated with sugars and negatively correlated with organic acids. The expression profiles indicated that these *CsWRKY*s had tissue-specific expression patterns (**Figures 5F, G**). Two *CsWRKY*s (*Cs2g_pb001850* and *Cs8g_pb012170*) are rarely expressed in fruit but highly expressed in root. Notably, the transcript abundance of two *CsWRKY*s (*Cs5g_pb002530* and *Cs4g_pb021230*) in young fruit was higher than that in mature fruit, while three *CsWRKY*s (*Cs6g_pb004840*, *Cs7g_pb004560*, and *Cs4g_pb011150*) showed higher expression levels in mature fruit. These 10 *CsWRKY*s may be candidate genes involved in sugar and organic acid metabolism.

### Expression analysis of *CsWRKY*s and determination of TSS and TA in orange fruit

**Figure 6:**
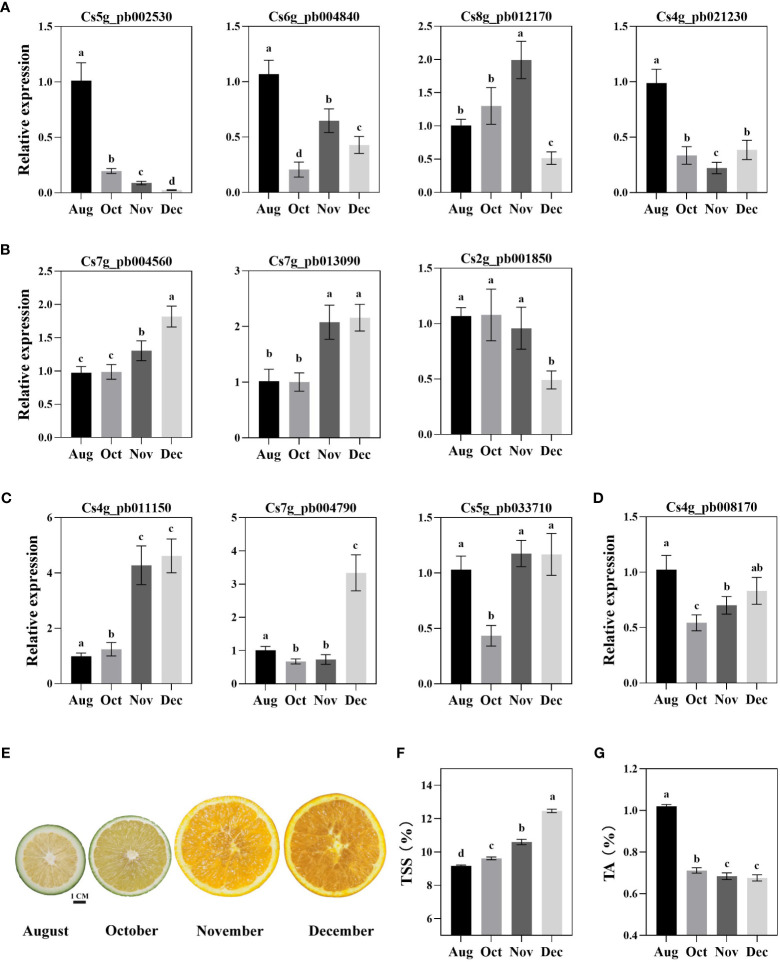
Expression of *CsWRKY*s and content of total soluble solid (TSS) and titratable acidity (TA) in the pulp of Newhall navel orange fruits at different development stages. Gene expression of *CsWRKY*s correlated with sugar **(A, B)** organic acid **(C, D)** in fruit pulp. The phenotype of orange fruit at development stages **(E)**. TSS **(F)** and TA **(G)** in the pulp of orange fruit. Lowercase letters indicate significant differences (*p* < 0.05) analyzed using Duncan’s test.

To further explore the potential function of *CsWRKY*s in the metabolism of sugars and organic acids, qRT-PCR analysis was performed on candidate *CsWRKY*s. The expression profiles indicated that two *CsWRKY*s (*Cs5g_pb002530* and *Cs4g_pb021230*) showed the highest transcription levels in August, while their expression dramatically decreased with fruit development. The expression of *Cs6g_pb004840* showed a fluctuating trend; meanwhile, the expression of *Cs8g_pb012170* increased with fruit development followed by a significant decrease in December (**Figure 6A**). Moreover, two candidate *CsWRKY*s positively correlated with sugars had different expression trends in the pulp of orange fruit. *Cs7g_pb004560* had an overall upregulated expression trend, while the expression of *Cs2g_pb001850* showed slight changes (**Figure 6B**). As shown in **Figure 6C**, the transcriptional level of *Cs4g_pb011150* increased with fruit development, *Cs7g_pb004790* showed the highest level in December, and *Cs5g_pb033710* showed the lowest level in October. *CsWRKY*s (*Cs2g_pb001850* and *Cs8g_pb012170*) positively correlated with organic acid and showed different expression patterns (**Figures 6A, D**). Furthermore, the main fruit quality, namely, TSS and TA, were determined at different stages of fruit development (**Figure 6E**). The TSS in fruit showed a consistently decreasing trend throughout the fruit development and reached approximately 12.5% in December (**Figure 6F**). Meanwhile, the TA content in fruit showed a significant decrease after August, followed by a slight decrease with fruit development (**Figure 6G**). Combining expression profile and fruit quality, three *CsWRKY*s (*Cs5g_pb002530*, *Cs7g_pb004560*, and *Cs4g_pb011150*) with high transcriptional abundance in fruit had strong correlations with fruit sugars or organic acids.

### Co-expression network analysis of candidate *CsWRKY*s during fruit development

**Figure 7:**
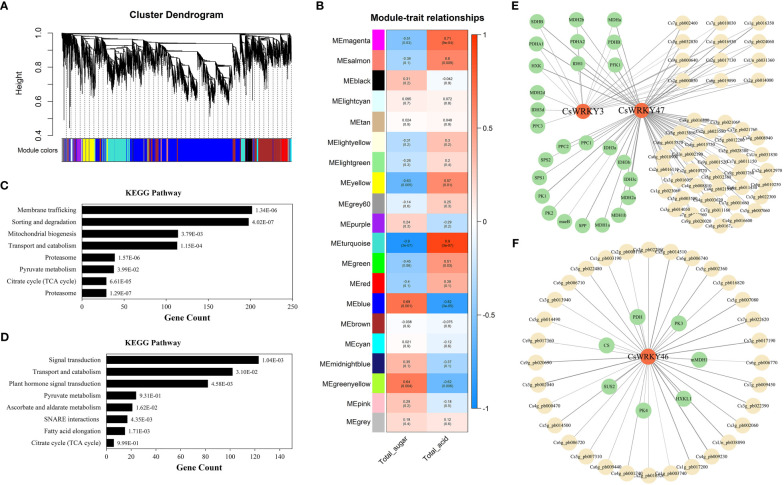
**(A)** Hierarchical clustering tree showing co-expression modules identified by WGCNA. Different modules are marked with different colors. Each leaf of the cluster tree represents a gene. **(B)** Module–trait correlations and corresponding *p*-values. TA and TSS. **(C, D)** KEGG pathway enrichment analysis of modules. **(E, F)** Co-expression network analysis of *CsWRKY*s. WGCNA, weighted gene co-expression network analysis; TA, titratable acid; TSS, total soluble solid; KEGG, Kyoto Encyclopedia of Genes and Genomes.

To further investigate the regulatory mechanism of candidate *CsWRKY*s, WGCNA was performed using the published transcriptome data of ‘Fengjie 72-1’ with five development stages. A total of 20 merged co-expression modules were finally identified (**Figure 7A**). Interestingly, the module in turquoise color (6,466 genes) was negatively associated with total sugar (correlation coefficient = −0.9) but positively associated with organic acid (correlation coefficient = 0.9). The module in blue color (4,871 genes) was positively associated with total sugar (correlation coefficient = 0.69) but negatively associated with organic acid (correlation coefficient = −0.82) (**Figure 7B**; **Table S4**). In addition, the candidate *CsWRKY*s (*Cs7g_pb004560* named *CsWRKY3* and *Cs4g_pb011150* named *CsWRKY47*) with an upregulated trend were found in blue modules, and *Cs5g_pb002530* named *CsWRKY46* with a downregulated trend was found in the turquoise module. Furthermore, Kyoto Encyclopedia of Genes and Genomes (KEGG) pathway enrichment analysis showed that most genes in the turquoise module were significantly enriched in various pathways (*p* < 0.05), such as Pyruvate metabolism (ko00620) and Citrate cycle (ko00020) (**Figure 7C**). Meanwhile, genes in the turquoise module were also significantly enriched in carbohydrate metabolism pathways, including Pyruvate metabolism (ko00620), Ascorbate and aldarate metabolism (ko00053), and Citrate cycle (ko00020) (**Figure 7D**; **Table S5**).

The co-expression networks further revealed that multiple genes involved in carbohydrate metabolism were closely linked to the candidate *CsWRKY*s (weight > 0.15). As shown in **Figure 7E** and **Table S6**, *CsWRKY3* was observed to co-express with *succinate dehydrogenase B* (*SDHB*), three *malate dehydrogenase* genes (*MDH2b*, *MDH2d*, and *MDHa*), three *pyruvate dehydrogenase* genes (*PDHA1*, *PDHB*, and *PDHA2*), *hexokinase* (*HXK*), and two *isocitrate dehydrogenase* genes (*IDH1* and *IDH3d*) *6-phosphofructokinase 1* (*PFK1*) and *phosphoenolpyruvate carboxylase 3* (*PPC3*). Meanwhile, there were three *PPC*s (*PPC1*, *PPC2*, and *PPC3*), two *sucrose-phosphate synthase* genes (*SPS1* and *SPS2*), two *pyruvate kinase* genes (*PK1* and *PK2*), four *IDH*s (*IDH1*, *IDH3a*, *IDH3b*, and *IDH3c*), five *MDH*s (*MDHa*, *MDH2b*, *MDH2a*, *MDH1a*, and *MDH1b*), *malate dehydrogenase B* (*maeB*), and *sucrose-6-phosphatase* (*SPP*). Among these carbohydrate metabolism genes, three *PDH*s, *HXK*, *IDH1*, *SDHB*, and two *MDH*s were shown to be linked to both *CsWRKY3* and *CsWRKY47*. Moreover, the downregulated *CsWRKY46* was found to co-express with two *PK*s (*PK3* and *PK4*), *PDH*, *HXKL1*, *mMDH1*, *sucrose synthase 2* (*SUS2*), and *citrate synthase* (*CS*) (**Figure 7F**). These results indicated that *CsWRKY*s participated in the modulation of fruit sugar and organic acid metabolism by regulating different genes during fruit development.

## Discussion

Citrus fruit is an economically important fresh fruit worldwide. The contents of sugars and organic acids in fruit are considered the crucial factor that determines fruit flavor and commodity value (Lin et al., 2015). To date, more and more studies have been conducted to investigate the regulatory mechanisms of sugar and organic acid metabolism at the transcriptional level. As one of the largest transcription factor families in higher plants, the WRKY gene family has been found to play an essential role in plant growth, development, and biotic and abiotic stresses (Eulgem et al., 2000; Rushton et al., 2010; Han et al., 2018b; Han et al., 2021a). However, there is still limited information on the functional CsWRKYs in the formation of citrus fruit quality.

### Characteristics of *CsWRKY* in citrus genome

Recently, genome-wide analysis of WRKY gene family has been reported in multiple plants, such as *A. thaliana*, tomato, tobacco, and rice (Kim and Zhang, 2004; Wu et al., 2005; Ross et al., 2007; Chen et al., 2015; Liu et al., 2022a). In the present study, a total of 81 *CsWRKY*s were identified in the *C. sinensis* genome, and the number was greater than that in other citrus genomes, which may account for more transcripts in the genome (Ayadi et al., 2016; Mosharaf et al., 2020). The sequence alignment and phylogenetic analysis showed that all *CsWRKY*s were divided into three groups and had the conserved N-terminal WRKYGQK domains and zinc finger-like motifs that were consistent with WRKY structures in other species (**Figures 2**, **3**; **Table S1**). As is known, these prominent features of the WRKY family directly establish their roles in gene regulation (Wei et al., 2016; Chen et al., 2021). In this study, all of the CsWRKY proteins contained at least one WRKY domain, whereas several of them lacked zinc finger-like motifs (**Figures 3A**, **S1**). Moreover, two *CsWRKY*s (*Cs7g_pb004560.2* and *Cs7g_pb004560.4*) in group I lost the second WRKY domains and zinc finger-like motifs, which may result in functional changes.

*Cis*-elements are the regulatory sites located at the 5′ upstream sequence of genes, and their variety always determines the diversity of regulatory functions of genes. In our results, *CsWRKY*s were found to be enriched in hormone-responsive and light-responsive *cis*-elements, as well as various abiotic-responsive *cis*-elements (**Figure 3C**; **Table S3**). The enrichment of different *cis*-elements in different *CsWRKY*s indicated their diverse functions involved in plant development and stress response. Notably, the ABA-responsive element was observed to exist in promoters of more than 60 *CsWRKY*s. As reported previously, ABA is an important phytohormone regulating plant growth and development (Yoshida et al., 2019). Multiple studies have demonstrated that ABA signaling is involved in various metabolic pathways that contribute to fruit growth and ripening (Liao et al., 2018; Feng et al., 2021; Zhu et al., 2023). Meanwhile, several WRKYs were also found to participate in ABA-regulated fruit development, such as VvWRKY22 (Huang et al., 2021). Herein, the enrichment of ABA-responsive elements in *CsWRKY*s indicated their functional roles in the formation of fruit quality and development of fruit. The mechanism of their responsiveness to ABA signals, as well as transcriptional regulation, deserves more study.

### CsWRKYs are involved in the formation of fruit quality in citrus fruit

With the development of citrus fruit, the content of total sugar showed an obvious upward trend, while the content of organic acid significantly reduced (**Figures 6F, G**). The trend of sugar and organic acid changes is a universal pattern in most citrus fruits (Li et al., 2017; Shi et al., 2019; Bi et al., 2022). As an important regulatory factor in plants, WRKY TFs have been investigated to play critical roles in fruit development, as well as the stress response (Han et al., 2020; Liu et al., 2022b; Guan et al., 2023; Shao et al., 2023). Meanwhile, transcriptome analysis of different tangor mandarins also revealed that multiple *WRKYs* had potential functions in fruit quality formation throughout fruit development (Bi et al., 2022; Liao et al., 2022). In this study, the expression profiles of WRKY TFs and fruit quality (sugars and organic acids) of orange fruit were conjointly analyzed and revealed that several of these genes may be involved in the regulation of fruit quality formation (**Figures 4**, **5**). Among them, three *CsWRKY*s with significant correlation with fruit sugars and organic acids, namely, *CsWRKY3*, *CsWRKY47*, and *CsWRKY46*, were regarded as candidate genes (**Figures 6**, **7**). Notably, ABA-responsive elements were also identified in the promoters of these *CsWRKY*s (eight in *CsWRKY3* and *CsWRKY47* and four in *CsWRKY46*) (**Table S3**). As the key components in ABA signaling, WRKYs participate in regulating ABA-induced responses to plant growth and stresses (Han et al., 2021a; Wang et al., 2023a). Therefore, our findings further indicated that the WRKY-regulated sugar/organic acid metabolism may be modulated by ABA.

Subsequent co-expression network analysis revealed that the genes co-expressed with fruit sugars and organic acids were enriched in different metabolism pathways (**Figures 7A, B**; **Table S5**). Many of these genes were further clustered into several carbohydrate metabolism pathways, including Glycolysis metabolism, Pyruvate metabolism, and Citrate cycle, which directly regulated the metabolism of sugars and organic acids (**Figures 7C, D**). Previous studies have revealed that the accumulation and degradation of sugar and organic acid components are influenced by the changes in transcriptional levels of carbohydrate metabolism genes (for example, *SUS*s, *PFK*s, *HXK*s, *CS*s, and *IDH*s) and are regulated by different TFs (for example, ZATs, HLHs, MYBs, and WRKYs) (Chen et al., 2017; Wei et al., 2019; Yu et al., 2021; Liu et al., 2022c; Yu et al., 2022; Fang et al., 2023). Herein, multiple genes (such as *IDH*s, *PDH*s, *HXK*s, *PK*s, *SPS*s, and *MDH*s) involved in carbohydrate metabolism were identified to be closely linked to *CsWRKY*s with the formation of fruit quality. Notably, the upregulated *CsWRKY*s (*CsWRKY3* and *CsWRKY47*) were found to co-express with most carbohydrate metabolism genes (**Figures 7E, F**). Similarly, *WRKY3* was reported to co-express with sugar-related genes and further promote the sucrose metabolism in *Arachis hypogaea* L. and *Hylocereus* (Han et al., 2018a; Kiranmai et al., 2018; Wei et al., 2019). However, most homologous genes of *WRKY47* in different plants were identified to participate in abiotic stress responses, such as selenium tolerance (Wu et al., 2020), boron tolerance (Feng et al., 2020), and drought response (Raineri et al., 2015). There is little information on the roles of WRKY47 in the formation of fruit quality. Hence, the candidate *CsWRKY*s identified in this study still need more studies, and the results will provide more information in analyzing the regulatory network of fruit quality.

## Conclusion

In the present study, a total of 81 *CsWRKY*s were identified in the *C. sinensis* genome. All *CsWRKY*s were classified into three major groups: I, II, and III. CsWRKYs of group II were further divided into five distinct subgroups: IIa, IIb, IIc, IId, and IIe. All identified CsWRKY proteins had the conserved WRKY domains and zinc-finger motifs. Furthermore, expression analysis revealed that multiple *CsWRKY*s co-expressed with sugars and organic acids during citrus fruit growth. Notably, three *CsWRKY*s, named *CsWRKY3*, *CsWRKY47*, and *CsWRKY46*, were identified as the key genes involved in the metabolism of fruit sugars and organic acids. The expression network further indicated that these candidate *CsWRKY*s may modulate the fruit quality by regulating carbohydrate metabolism genes. Our results provided new insights into the regulatory mechanism of *CsWRKY*s in the formation of fruit sugars and organic acids.

## Data availability statement

The datasets presented in this study can be found in online repositories. The names of the repository/repositories and accession number(s) can be found in the article/**Supplementary Material**.

## Author contributions

MZ: Conceptualization, Funding acquisition, Investigation, Resources, Validation, Writing – review & editing. WL: Formal Analysis, Investigation, Writing – original draft. XY: Investigation, Resources, Validation, Writing – original draft. QL: Investigation, Resources, Writing – original draft. XL: Investigation, Writing – original draft. KL: Investigation, Writing – original draft. CY: Investigation, Writing – original draft. BX: Resources, Supervision, Writing – review & editing. LL: Resources, Supervision, Writing – review & editing. GS: Resources, Supervision, Writing – review & editing. SH: Resources, Supervision, Writing – review & editing. JH: Resources, Supervision, Writing – review & editing. XW: Conceptualization, Funding acquisition, Writing – review & editing. ZW: Conceptualization, Funding acquisition, Writing – review & editing.
