# Supplementary figures and images for: Comprehensive analyses of the citrus WRKY gene family involved in the metabolism of fruit sugars and organic acids
Source: Front Plant Sci. 2023 Sep 15;14:1264283. doi: 10.3389/fpls.2023.1264283 (PMC10540311; doi:10.3389/fpls.2023.1264283)

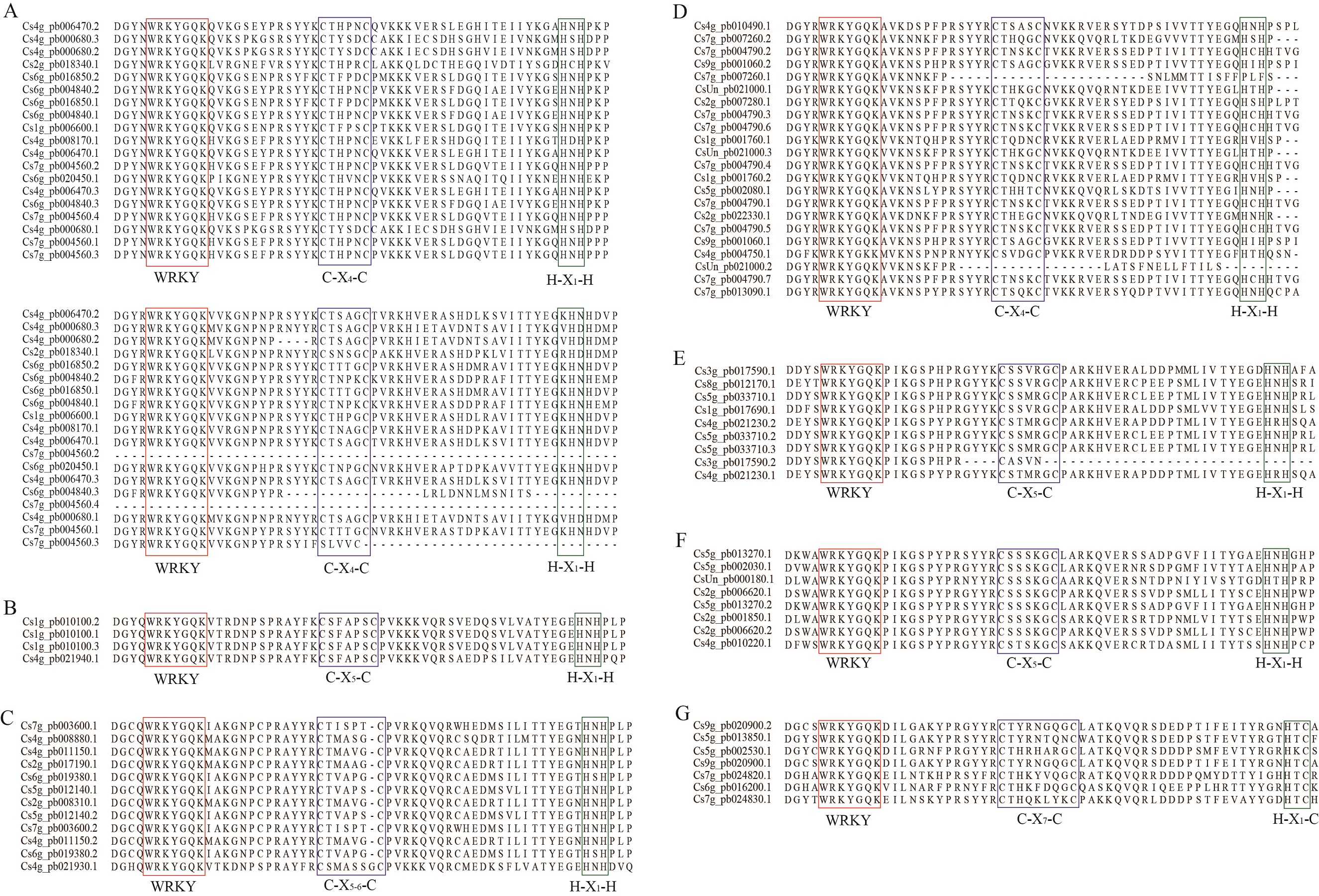

Supplement: Supplementary file 1 [file Image_1.jpeg]
